# Born Too Soon: Progress, priorities and pivots for preterm birth

**DOI:** 10.1186/s12978-025-02067-1

**Published:** 2025-06-23

**Authors:** Tedros Adhanom Ghebreyesus, Natalia Kanem, Catherine Russell, Helen Clark

**Affiliations:** 1 World Health Organization (WHO), Geneva, Switzerland; 2 United Nations Population Fund (UNFPA), New York, USA; 3 United Nations Children’s Fund (UNICEF), New York, USA; 4 Partnership for Maternal, Newborn, Child Health (PMNCH), World Health Organization (WHO), Geneva, Switzerland

Where you are born, or how much money your family has, should not determine whether you live or die. However, despite progress in the last decade, this is still the reality for many mothers and babies, especially babies who are born too soon.

The 2012 Born Too Soon report ignited a movement to improve care for preterm babies [1]. The partnership included stakeholder groups ranging from the grassroots to high-level political champions. The result was accelerated action for maternal and newborn health, including targets in the Sustainable Development Goals (SDGs) on maternal and newborn survival and linked action plans (the Every Newborn Action Plan and the Ending Preventable Maternal Mortality Strategy) [2]. The movements for maternal and newborn health have come together under our respective entities, recognising the need for stronger advocacy and better integrated services for women and newborns.

A decade on, an even larger coalition of partners is uniting through this new version of Born Too Soon to call for a healthier and more equitable future for millions of babies, women and families [3]. Much has changed in the past ten years, with new evidence, updated tools and guidance, and inspiring success stories and innovations. Yet, despite this, preterm birth rates continue to raise much concern, with around a million preterm babies dying each year [4]. This signals that we are failing on primary prevention: interventions directed at all women during pregnancy, particularly in the antenatal period, to ensure a healthy pregnancy and detect risks early on. Complications from preterm birth remain the leading cause of death among newborns, the period where mortality is the highest among children [4].

The devastating loss to individual mothers and families in every country is a sorrow never forgotten. The loss to national development and human capital is incalculable. What is more, when progress seems possible, efforts have been knocked back by conflicts, the climate crisis, the COVID-19 pandemic, the economic downturn and other adverse events.

Our children are our future. Their right to health, wellbeing and life is the cornerstone of healthy and productive families and societies. As leaders of United Nations agencies and of the largest global alliance for women’s, children’s, and adolescents’ health, we are committed to accelerating progress. This requires working across sectors, in education, and improving the quality of care and financial protection, in the spirit of the SDGs. Most importantly, it requires strong national and sub-national leadership and bottom-up mobilisation of stakeholders, including civil society and communities.

We are proud that our respective organisations, along with countless individuals and partners, have joined efforts to produce this report and commit to the actions it outlines. Together, we have all the ingredients for sustained progress, and many countries around the world, showcased in this report, have shown how it can be achieved.

The next generation depends on us acting now.

## Data Availability

All data is available in the paper or in supplementary files. Additional information is available at www.borntoosoonaction.org.
